# Proteogenomics Reveals Perturbed Signaling Networks in Malignant Melanoma Cells Resistant to BRAF Inhibition

**DOI:** 10.1016/j.mcpro.2021.100163

**Published:** 2021-10-19

**Authors:** Marisa Schmitt, Tobias Sinnberg, Katrin Bratl, Katharina Zittlau, Claus Garbe, Boris Macek, Nicolas C. Nalpas

**Affiliations:** 1 Quantitative Proteomics, University of Tuebingen, Tuebingen, Germany; 2 Division of Dermatooncology, University of Tuebingen, Tuebingen, Germany; 3 Cluster of Excellence iFIT (EXC 2180), Image-Guided and Functionally Instructed Tumor Therapies, University of Tuebingen, Tuebingen, Germany

**Keywords:** proteogenomics, cancer, melanoma, BRAFi resistance, phosphorylation, mass spectrometry, nucleotide sequencing, A375 R, BRAFi-resistant A375 cells, A375 S, BRAFi-sensitive A375 cells, AGC, automatic gain control, AURKA, aurora kinase A, AURKAi, AURKA inhibition, BRAF, serine/threonine-protein kinase B-raf, BRAFi, BRAF inhibitor, DHB, dihydroxybenzoic acid, ERK, extracellular signal-regulated kinase, FBS, fetal bovine serum, FDR, false discovery rate, HCD, higher-energy collisional dissociation, LC-MS/MS, liquid chromatography–tandem mass spectrometry, MAPK, mitogen-activated protein kinase, MS, mass spectrometry, NOG, N-ocetylglucoside, PTM, posttranslational modification, RT, room temperature, RUNX1, runt-related transcription factor 1, SNV, single nucleotide variant, WES, whole-exome sequencing

## Abstract

Analysis of nucleotide variants is a cornerstone of cancer medicine. Although only 2% of the genomic sequence is protein coding, mutations occurring in these regions have the potential to influence protein structure or modification status and may have severe impact on disease aetiology. Proteogenomics enables the analysis of sample-specific nonsynonymous nucleotide variants with regard to their effect at the proteome and phosphoproteome levels. Here, we developed a proof-of-concept proteogenomics workflow and applied it to the malignant melanoma cell line A375. Initially, we studied the resistance to serine/threonine-protein kinase B-raf (BRAF) inhibitor (BRAFi) vemurafenib in A375 cells. This allowed identification of several oncogenic nonsynonymous nucleotide variants, including a gain-of-function variant on aurora kinase A (AURKA) at F31I. We also detected significant changes in abundance among (phospho)proteins, which led to reactivation of the MAPK signaling pathway in BRAFi-resistant A375 cells. Upon reconstruction of the multiomic integrated signaling networks, we predicted drug therapies with the potential to disrupt BRAFi resistance mechanism in A375 cells. Notably, we showed that AURKA inhibition is effective and specific against BRAFi-resistant A375 cells. Subsequently, we investigated amino acid variants that interfere with protein posttranslational modification (PTM) status and potentially influence A375 cell signaling irrespective of BRAFi resistance. Mass spectrometry (MS) measurements confirmed variant-driven PTM changes in 12 proteins. Among them was the runt-related transcription factor 1 (RUNX1) displaying a variant on a known phosphorylation site S(Ph)276L. We confirmed the loss of phosphorylation site by MS and demonstrated the impact of this variant on RUNX1 interactome.

## Highlights

• Proteogenomics workflow to study the malignant melanoma cell line A375.
• Key signaling pathways are perturbed in BRAFi-sensitive and -resistant A375.
• The signaling network in BRAFi-resistant cells can be targeted by several drugs.
• Multiple amino acid variants directly affect protein phosphorylation status.
• Loss of a phosphorylation site on RUNX1 alters its interactome.

## Graphical Abstract

**Figure:**
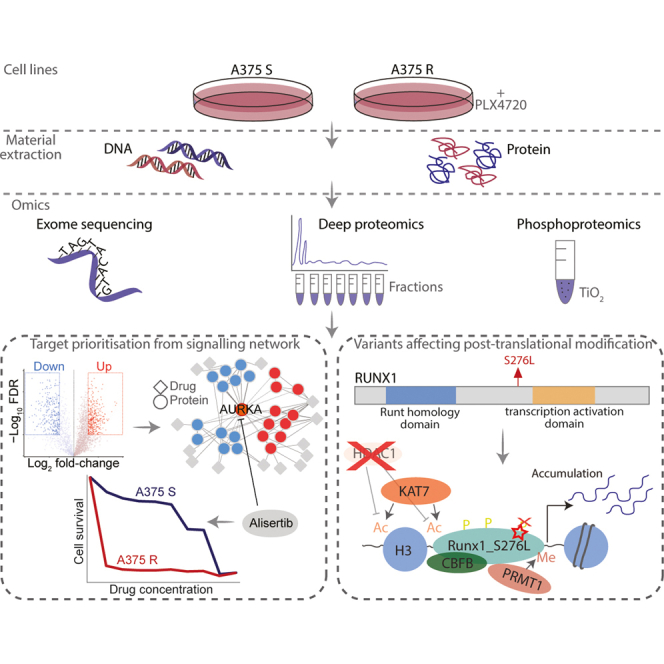

## In Brief

Proteogenomics is a powerful tool to study the mode of action of disease-associated mutations at the genome, transcriptome, proteome, and PTM level. Here, we applied a proteogenomics workflow to study the malignant melanoma cell line A375. Such workflow, used here as a proof of concept on A375 cells, may be applicable to other cancer types, cell lines, or even patient-derived samples.

Accumulation of mutations is one of the hallmarks of cancer cells, and malignant melanoma is a type of cancer with the highest frequency of somatic mutations (1). Recent investigations showed that mutations of key signaling pathways in malignant melanoma are associated with poor clinical outcome, for example, in the mitogen-activated protein kinase (MAPK)/extracellular signal-regulated kinase (ERK) pathway (2). The RAS/BRAF/MEK/ERK pathway is mutated to an oncogenic form in 30% of all cancers, with nonsynonymous BRAF mutations in up to 50% of cutaneous melanomas (3). The predominant BRAF mutation is within the kinase domain with a single nucleotide substitution of valine to glutamic acid at amino acid 600 (4). This mutation can result in a 500-fold increased, dimerization-independent activation of BRAF and thus leads to a constitutive activation of downstream signaling in cancer cells (3, 5). Targeted inhibition of the mutated BRAF kinase with selective inhibitors such as vemurafenib, dabrafenib, or encorafenib (BRAFi) results in a reduction of MAPK pathway signaling (5). However, almost all patients rapidly develop resistance to BRAFi monotherapy after a period of approximately 5 months (2). The considerable majority of BRAF resistance development is caused by molecular or genetic alterations that lead to MAPK pathway reactivation. The identification of multiple cellular mechanisms of resistance has greatly improved the understanding of malignancy and clinical outcomes of BRAF^V600E^ metastatic melanoma, *e.g.*, by the introduction of combined BRAF and MEK inhibition.

The past decade has seen a revolution in high-throughput sequencing technologies, which provide information on DNA/RNA sequence, gene structure and expression (6). Mass spectrometry (MS)-based proteomics is experiencing a technological revolution similar to that of the high-throughput sequencing. The current state-of-the-art “shotgun” proteomics workflows are capable of routine, comprehensive analysis of proteomes (7, 8) and posttranslational modifications (PTMs) such as phosphorylation (9, 10). In recent years, such workflows have been combined with high-throughput sequencing technologies to investigate colon and rectal cancer (11), breast cancer (12, 13), squamous cell lung cancer (14) and lung adenocarcinoma (15). Such proteogenomic approaches have proved superior to interpret nucleotide variants in context of cellular signaling, phenotype, patient heterogeneity, and therapy prediction. In addition, proteogenomics allows the integration of sample-specific nonsynonymous variants into reference protein sequence database, thus enabling detection and quantification of amino acid variants by MS. For example, this permits the study of variants that alter the protein modification status (12, 16). In recent years, the number of human protein–protein interaction has considerably increased in the literature (17); these are now supplemented with clinical knowledge to deliver on precision medicine (18). Several studies have demonstrated the relevance of protein–protein network reconstruction to investigate network-attacking mutations (16), identify genomic alterations for therapeutic combinations (19, 20) or determine novel targets from differential networks (21).

Here we applied a proteogenomics approach to a single immortalized human melanoma cell line, A375, in its parental as well as in BRAFi-resistant state. We identified nonsynonymous nucleotide variants and quantified (phospho)proteins to reconstruct the signal transduction networks in context of acquired resistance to kinase inhibitors within A375 cells. We were able to prioritize a number of drugs based on their disruptive potential on this signal transduction networks. Finally, we investigated the impact of nonsynonymous amino acid variants on protein phosphorylation sites. And their putative functional effects were evaluated on A375 cell signaling irrespective of BRAFi resistance status.

## Experimental Procedures

Only star methods are presented below; the rest of the methods are described fully in supplemental data.

### Experimental Design and Statistical Rationale

For the whole-exome sequencing (WES), DNA was extracted from A375 sensitive (S) and resistant (R) cells. Since WES was used only for variant calling, one replicate was analyzed for each cell line. Total genomic DNA was enriched for exome regions and sequenced on Illumina HiSeq 2000. Obtained reads were aligned to *H. sapiens* reference genome (GRCh38) using the HiSAT2 aligner. Variants were called using GATK software and incorporated into cell-line-specific protein sequence database using in-house script.

The (phospho)proteomics data is derived from two sets of samples prepared and analyzed by liquid chromatography–tandem mass spectrometry (LC-MS/MS). For the first screen, a total of 114 runs were performed with 60 min gradient for fractionated proteome and phosphoproteome measurements on an Exploris mass spectrometer. A375 S and A375 R cells were used for proteome and phosphoproteome measurements (three biological replicates per cell line, with 19 samples per replicate consisting of nine fractionated proteome samples and ten rounds of phosphopeptide enrichment). For the second screen, a total of 21 runs were performed with 60 min gradient for immunoprecipitation and 90 min gradient for peptide pull-down measurements on Q Exactive HF-X and HF mass spectrometers. Immunoprecipitation assays of Flag-tagged RUNX1, SILAC labeled A375 S RUNX1_KO cells transfected with pCMV_Flag_RUNX1 plasmid, pCMV_Flag_RUNX1_S276L plasmid, or with empty vector plasmid (pCMV_Flag) were used (“light”: “medium”: “heavy”) (three biological replicates). For synthetic peptide pull-downs in A375 S cells, label-free quantification between three independent replicates was performed (nine samples). Beads only were used as a control (three replicates).

LC-MS/MS raw data were processed using MaxQuant software (version 1.6.8.0 and 1.5.2.8). Statistical analyses were performed with Perseus (version 1.6.0.5) for the (phospho)proteome datasets (*t* test and Fisher exact test).

Biological assays were performed in three biological and six technical replicates, so that appropriate statistical analysis could be performed. Statistical analysis was performed with two-tailed unpaired *t* test in GraphPad Prism (version 8). Separate controls were included in each experiment.

### Cell Culture

The human metastatic BRAF^V600E^-mutated melanoma cell line A375 (CRL-1619, ATCC) was used in this study and authenticated by Microsynth AG. The generation of the cell line with acquired resistance to vemurafenib analogue PLX4720 (Selleckchem) (for simplicity referred to as “vemurafenib” in the Results section) was conducted as described previously (22). A375 S and R cells were grown in RPMI medium (Sigma-Aldrich) supplemented with foetal bovine serum (FBS, 10%, PAN Biotech) and penicillin/streptavidin (100 U/ml, PAN Biotech) at 37 °C and 5% CO_2_.

For immunoprecipitation assays, SILAC labeling of cells was performed as described previously (23) and detailed description of labeling of cells, CRISPR/Cas9-mediated knockout of RUNX1, and interaction assays can be found in the supplemental data.

### Incorporation of Nonsynonymous Variants into Protein Databases

To integrate the proteogenomics datasets, we used an in-house bioinformatics pipeline, which is coded entirely in the R programming language (24). The transcript nucleotide sequences were extracted from GRCh38 *H. sapiens* genome assembly and Ensembl transcript annotation (*via* BSgenome and GenomicFeatures packages). These sequences were then *in silico* translated (from start to first stop codon) into a reference protein sequences database (Biostrings package). The called variants, within Variant Call Format files from A375 R and A375 S, were injected into each overlapping reference transcript nucleotide sequences and then *in silico* translated. The resulting protein sequences were written into two FASTA files containing reference variant protein sequences and sample-specific alternate variant protein sequences.

### Annotation of the Biological Impact of Detected Variants

In the current study, we prioritized amino acid variants based on their impact in context of BRAFi resistance in melanoma (supplemental Tables S1 and S2). For this purpose, known variant sites in melanoma, as well as known variant sites in cancer, were obtained from CGDS (25). These were overlapped with A375 identified variants and classified as loss/gain of sites. A list of oncogenes and tumor suppressor genes was compiled from Cosmic, ONGene, Bushman lab, and Uniprot (26, 27, 28), whereas a list of genes harboring variants involved in BRAFi-resistant tumor was retrieved after reanalysis of published study (29). A375 variants found on these genes were annotated as relevant in cancer and/or BRAFi resistance.

A second impact scoring strategy was also performed to investigate protein phosphorylation-based signal transduction networks in A375 melanoma cells. Each reference/alternate variant protein sequence was annotated based on whether phosphorylation sites (S/T/Y) were lost and/or gained (IRanges package). A list of known kinase motifs was retrieved from PhosphoNetworks (30) and these motifs were searched along the reference/alternate variant protein sequences. Located kinase motifs were overlapped with variants position to determine loss/gain of the motifs. Known human phosphorylation sites were retrieved from PhosphoSitePlus and Phospho.ELM databases (31, 32). The variants identified in our study, which overlapped with known phosphorylation sites, were annotated as loss/gain of known phosphorylation. In a similar fashion, known variant sites in melanoma were obtained from CGDS (25) and overlapped with the variants from A375 R and S. A list of oncogenes and tumor suppressor genes was compiled from Cosmic, ONGene, Bushman lab, and Uniprot (26, 27, 28). Variants on these genes that were identified in A375 R and S were annotated as cancer-relevant. A Levenshtein similarity score was calculated between reference and alternate variant protein sequences, whereby alternate sequences with less than 90% similarity to their reference were flagged.

Each amino acid within variant protein sequences was attributed a “+1” score for every overlap with an impact annotation. A summed score was then calculated for each amino acid within alternate variant sequence, and the maximum summed score was reported for that variant protein isoform. Because the score depends on the number of impacts used during the annotation, we also computed a scaled maximum score (between 0 and 1), to allow comparison between processings. Following the computation of all impacts, each variant protein isoform is ranked to allow prioritization for follow-up studies.

### Extraction and Digestion of Proteins

Cells were harvested at 80% confluence with lysis buffer (6 M urea, 2 M thiourea, 60 mM Tris pH 8.0) complemented with protease (complete Mini EDTA-free tablets, Roche) and phosphatase inhibitors (5 mM glycerol-2-phosphate, 5 mM sodium fluoride, and 1 mM sodium orthovanadate) and 1% N-ocetylglucoside (NOG, Sigma-Aldrich) for 10 min on ice. DNA and RNA were removed from the cell lysate using benzonase (1 U/ml, Merck Millipore) for 10 min on room temperature (RT). Cell debris was cleared by centrifugation (2800*g*, 10 °C, 20 min). Proteins were precipitated from cell lysates using eight volumes of acetone (−20 °C) and one volume of methanol and incubated overnight at −20 °C. The resulting solution was centrifuged (2800*g*, 10 °C, 20 min) to form a cell pellet. The pellet was washed two times with 80% acetone (−21 °C) and resuspended in lysis buffer without NOG. Protein concentration was measured using Bradford assay. Extracted proteins (2–3 mg) were reduced with 10 mM of dithiothreitol (DTT) for 1 h, alkylated with 55 mM iodoacetamide for an additional hour, and digested with Lys-C (Lysyl Endopeptidase, Wako Chemicals) for 3 h at RT. After adding four volumes of 10 mM ammonium bicarbonate, proteins were digested with trypsin (Promega Corporation) overnight. To stop the digestion, 1% trifluoroacetic acid (TFA) was added.

Detailed description of high-pH reverse-phase chromatography can be found in the supplemental data.

### Phosphopeptide Enrichment

Enrichment of phosphorylated peptides was performed using TiO_2_ beads (Titansphere, 10 μm, GL Sciences). TiO_2_ beads were resuspended in DHB solution (80% ACN, 1% TFA, 3% 2,5-dihydroxybenzoic acid (DHB)) and incubated for 20 min. Digested peptides were purified using Sep-Pak C18 Cartridge (Waters). In brief, Sep-Pak C18 Cartridges were activated with methanol and washed two times with 2% ACN, 1% TFA. After loading the sample, the column was washed again with solvent A (0.1% TFA) and eluted with 80% ACN, 6% TFA. Purified peptides were added to the TiO_2_ beads (beads to protein ratio, 1:2) and incubated for 10 min for each enrichment round (10 enrichment rounds). Phosphopeptide-bound TiO_2_ beads were sequentially washed with 30% ACN, 1% TFA, followed by 50% ACN, 1% TFA, and 80% ACN, 1% TFA. Peptides were eluted with 5% NH_4_OH into 20% TFA followed by 80% ACN in 1% FA. The eluate was reduced by vacuum centrifugation, pH was adjusted to <2.7 with TFA, and peptides were desalted on C18 StageTips prior LC-MS/MS measurements.

### Liquid Chromatography–Mass Spectrometry

Peptides were measured on an EASY-nLC 1200 ultrahigh-pressure system (Thermo Fisher Scientific) coupled to a quadrupole Orbitrap mass spectrometer (Q Exactive HF and HFX and Exploris 480, Thermo Fisher Scientific) *via* a nanoelectrospray ion source. About 500 ng of peptides was loaded on a 20-cm analytical HPLC-column (75 μm ID PicoTip fused silica emitter (New Objective); in-house packed using ReproSil-Pur C18-AQ 1.9-μm silica beads (Dr Maisch GmbH)). LC gradient was generated by solvent A (0.1% FA) and solvent B (80% ACN in 0.1% FA) and 200 nl/min. Column temperature was kept at 40 °C. For screen 1, all samples were measured on an Exploris 480 mass spectrometer using 60 min gradient for fractionated proteome (optimized gradient for each fraction) and phosphoproteome samples. The mass spectrometer was operated in data-dependent mode, collecting MS spectra in the Orbitrap mass analyzer (60,000 resolution, 300–1750 *m/z* range) with an automatic gain control (AGC) set to standard and a maximum ion injection time set to automatic. For higher-energy collisional dissociation (HCD), the 20 most intense peptides were selected and fragments with a normalized collision energy of 28. MS/MS spectra were recorded with a resolution of 30,000 (fill time set to automatic). Dynamic exclusion was turned on and set to 30 s. An *m/z* inclusion list (tolerance 10 ppm) was used to increase peptide coverage for RUNX1 and AURKA within the proteome and phosphoproteome measurements.

For the second screen, analysis of RUNX1 overexpression interactome (measured on Q Exactive HF-X), full MS were acquired in the range of 300 to 1750 *m*/*z* at a resolution of 60,000 (fill time 20 ms, AGC target 3E6). Twelve most abundant precursor ions from a survey scan were selected for HCD fragmentation (fill time 110 ms), and MS/MS spectra were acquired at a resolution of 30,000 on the Orbitrap analyzer. Precursor dynamic exclusion was enabled with a duration of 20 s (AGC target 1E5). Synthetic peptide pull-downs were analyzed on Q Exactive HF mass spectrometer, full MS were acquired in the range of 300 to 1650 *m*/*z* at a resolution of 60,000 (fill time 25 ms, AGC target 3E6). MS/MS scans were acquired for the top seven most abundant precursor ions with a resolution of 60,000 and a fill time of 110 ms (AGC target 1E5).

### Mass Spectrometry Data Processing

The raw data files were processed with the MaxQuant software suite (version 1.6.8.0 and 1.5.2.8) (33). The Andromeda search engine searched MS/MS data against *H. sapiens* reference (GRCh38 Ensembl release 97; 99,354 entries) and cell-line-specific alternate databases (GRCh38 Ensembl release 97; 29,104 entries), as well as UniProt *H. sapiens* (UniProt release 2019/12; 95,943 entries) database and commonly observed contaminants. Carbamidomethylation of cysteine (C) was set as fixed modification and oxidation of methionine, phosphorylation at serine, threonine, or tyrosine were defined as variable modifications. Trypsin/P was selected as a protease. No more than two missed cleavages were allowed. The MS tolerance was set at 4.5 ppm and MS/MS tolerance at 20 ppm for the analysis using HCD fragmentation method. The false discovery rate (FDR) for peptides and proteins was set to 1%. For label-free quantification, a minimum of one peptide was required. For quantification of proteins in the immunoprecipitation experiments, the amino acids (Lys4)/(Arg6) and (Lys8)/(Arg10) were defined as “medium” and “heavy” labels for the comparison of RUNX1 overexpressed cell lines. For all other parameters, the default settings were used.

### Significance Testing and Pathway Analysis

Statistical analyses were performed with Perseus software suite (version 1.6.5.0). For the (phospho)proteome investigation of BRAFi resistance in A375 cells, the drug-sensitive (n = 3) and drug-resistant (n = 3) A375 cells were compared using (1) label-free quantification for the proteins and (2) intensities for the phosphorylation sites. Each omics dataset was analyzed separately and entries were filtered out if not quantified in all samples. Additionally, the reverse and potential contaminants were filtered out from the protein and phosphorylation site datasets. Notably, the phosphorylation sites' intensities were normalized by the corresponding proteins intensities. A *t* test was used to compute *p*-values and identify significantly changing entries between A375 R and S samples. The *p*-value was corrected for multiple testing with a permutation-based FDR (s0 = 0.1 and FDR ≤0.05 [proteome] or FDR ≤0.1 [phosphoproteome]). The proteins and phosphorylation sites, which were statistically tested in Perseus, are listed in supplemental Table S1.

For proteomic interaction studies of RUNX1, protein groups were kept for further statistical analysis only if quantified in three out of three replicates. The SILAC ratios of the three independent replicates were averaged and an arbitrary cutoff of twofold change was used to determine significant SILAC ratios. The log_2_-transformed ratios were plotted against intensities (log_10_). For synthetic peptide pull-downs, label-free quantification between three independent replicates was performed and ratios were subjected to *t* test analysis, with a permutation-based FDR threshold of 0.01 and s0 value of 1.2. A list of known interaction partners of RUNX1 was retrieved from BioGrid and mapped to the dataset. A list of all protein identifications is provided in supplemental Table S3.

The resources used for annotation of proteins were Kyoto Encyclopaedia of Genes and Genomes (KEGG), Gene Ontology Biological Function (GOBP), and Reactome Pathway database (Reactome). The Fisher exact test (FDR ≤0.2 [BRAFi resistance studies] or FDR ≤0.1 [interaction studies]) was used to test for overrepresented functions or pathways among significantly changing entries against the background of identified entries. The displayed pathways were selected based on highest FDR or enrichment score. A list of all overrepresentation results is provided in supplemental Tables S1 and S3.

### Amino Acid Variants Identification

Our in-house proteogenomics bioinformatic pipeline was used to integrate WES and MS datasets, specifically to check which mutations were identified across omic datasets. Initially, the reference and alternate variant protein sequences were *in silico* digested according to laboratory condition; *i.e.*, digestion with trypsin and up to two missed cleavages (cleaver package). The overlap of MS-identified peptides with *in silico* digested peptides led to classification into reference (nonmutated peptide that overlaps the mutation position on the reference protein), alternate (mutated peptide that overlaps the mutation position on the alternate protein), or unspecific (nonmutated peptide that does not overlap any mutated positions) variant peptides. On the basis of this peptide classification, we summarized the peptides identification per variant protein isoforms, allowing coverage characterization into reference only, alternate only, reference, and alternate or unspecific. We finally focused on PTM (as implemented in the MaxQuant processing), which here consists of phosphorylation sites. Reference and/or alternate variant peptides found phosphorylated were flagged as such, as well as those where the phosphorylation occurred directly on the variant sites (either on reference or alternate variant sequences). This coverage information is exported within MaxQuant style processing results (tab-separated file as output).

### Signaling Network Reconstruction

We downloaded the protein–protein interactions from the BioGRID database (release 3.5.169) (17) and reconstructed signaling network in the R programming language. We used only interactions that were reported in *H. sapiens* and showed at least two experimental evidences (*e.g.*, two publications, two methods). Networks were generated undirected, as such information is missing from BioGRID. In addition, self-linked interactions and orphan nodes were removed (igraph package). Nodes were organized relative to each other based on betweenness centrality and variant effect (*e.g.*, not mutated, loss of function, gain of function) for the network of BRAFi-resistant A375 or functional pathway for RUNX1 interactome network. For the network of BRAFi-resistant A375 cells, we retrieved interactions strictly between the significantly changing (phospho)proteins (between A375 S and R), as well as the list of proteins with potential driver mutations (*i.e.*, damaging oncogenic variants and variants unique to A375 R). Whereas, for the networks of AURKA and RUNX1, their respective interactors were retrieved irrespective of their identification by MS or WES. For protein target prioritization, we ranked (from high to low) the nodes based on number of edges within each interaction network and retained the top 200, 50, and 55 nodes for the network of BRAFi-resistant A375, AURKA, and RUNX1, respectively. The generated networks were exported (using igraph and RCy3 packages) into Cytoscape to improve visual formatting (34).

Possible drugs interacting with the network of BRAFi-resistant A375 cells were retrieved from DrugBank database (release 24.10.2019) based on their targets (35). Only drugs showing an effect in *H. sapiens* were used. All drugs were retained, irrespective of their category (*e.g.*, inhibitor), chemical kingdom (*e.g.*, organic compound), or approval status (*e.g.*, approved, experimental). The specificities of the drugs, interacting with nodes from the generated network, were calculated based on all possible target reported in DrugBank database. Drugs were prioritized further by summing the number of interactions their targets have within the network.

## Results

To study signal transduction networks using a proteogenomic approach, we selected the widely studied A375 melanoma cell line that harbors the BRAF^V600E^ mutation. We generated two closely related A375 cell lines, drug-sensitive (A375 S) and drug-resistant against the BRAF inhibitor vemurafenib (A375 R), as described previously (22). The resulting cell lines were subjected to WES, as well as proteomics and phosphoproteomics evaluation (supplemental Fig. S1*A*). These multiomic measurements were then integrated (1) to reconstruct signaling networks that are disturbed in BRAFi-resistant A375 cells and (2) to investigate the impact of variants altering protein PTM status in A375 cells irrespective of BRAFi resistance.

### Variance in Protein Abundance Discriminates between BRAFi-sensitive and -resistant A375 Cells

**Fig. 1:**
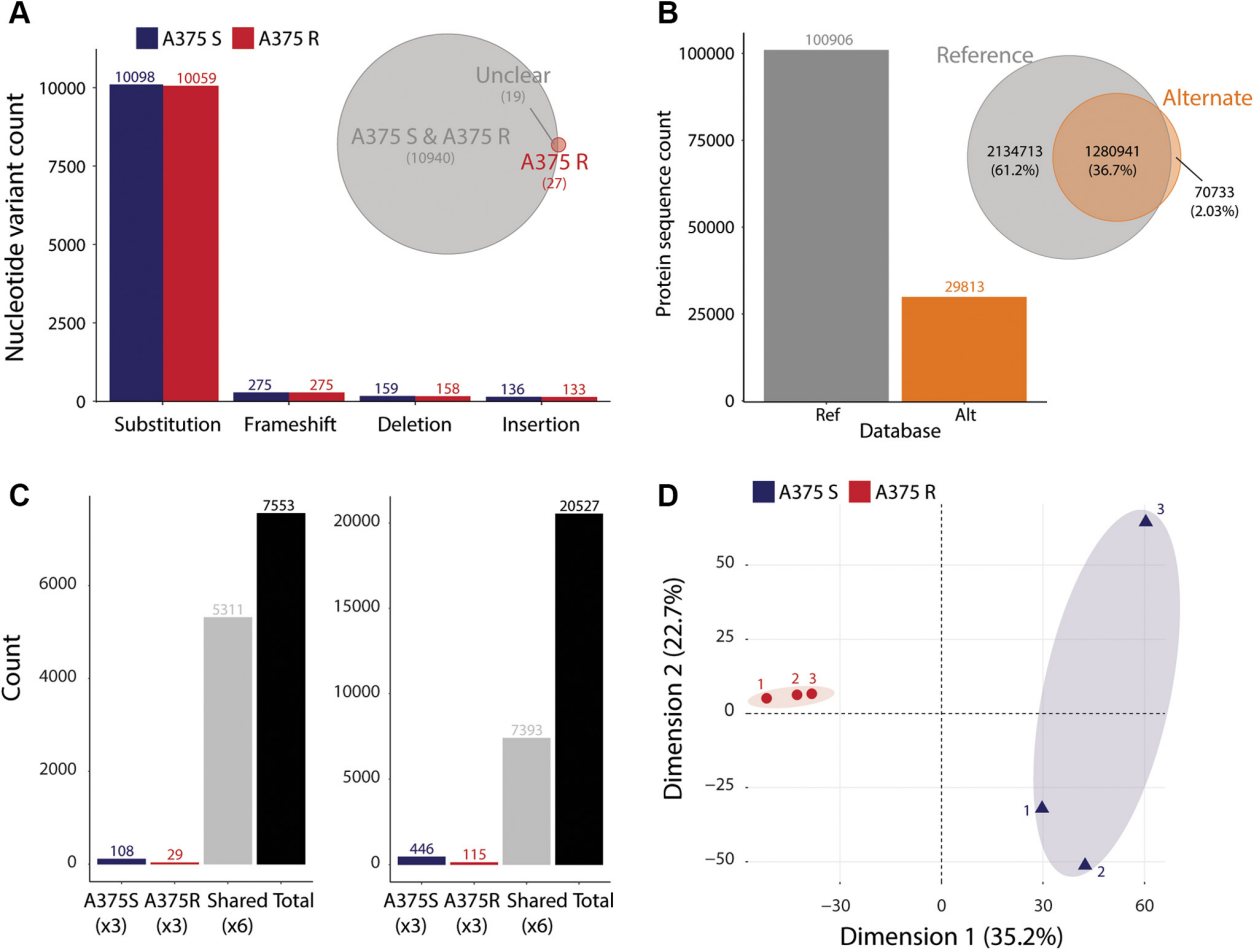
**Multiomics identification using proteogenomics.***A*, number of nonsynonymous nucleotide variants is classified per type of alteration for A375 S and R cells. The Venn diagram represents the overlap between A375 S and R shared variants and A375 R unique nonsynonymous nucleotide variants. *B*, the number of protein sequences is displayed per reference (ENSEMBL) or alternate (WES from A375 R and S) databases, as well as the overlap in search space between databases (up to two missed cleavages). *C*, quantified protein groups and phosphorylated sites are counted based on whether they are shared or unique between A375 R and S cell lines. *D*, principal component analysis using protein abundances shows the separation of samples between cell lines (A375 R *versus* S), as well as the clustering of the biological replicates. *Numbers* next to each dot indicate the replicate identifier.

Initially, we investigated the mutational landscape of A375 R and A375 S cells by high-throughput sequencing. The majority (95%) of nonsynonymous nucleotide variants consisted of nucleotide substitutions, the rest comprising frameshifts, deletions, and insertions (Fig. 1*A* and supplemental Table S1.1). This trend was consistent across both A375 cell lines. As expected, only 46 variants (out of 10,986) were classified as unique to A375 R cells, the rest being shared in both cell lines. Notably, most variants were already reported in Cosmic and/or dbSNP databases (supplemental Fig. S1*B*), allowing for functional effect annotation. The variants affected genes, and in turn the corresponding proteins, from every subcellular compartment, such as cytoplasm (1067 variants), nucleus (680), mitochondrial matrix (229), mitochondrial import complex (115), and mitochondrial outer membrane (36). Analysis of the reference to alternate nucleotide substitution revealed a high frequency (64% of all substitutions) of adenine to guanine (and vice versa), as well as cytosine to thymine (and vice versa) exchange (supplemental Fig. S1*C*). Subsequently, these nonsynonymous variants were incorporated into their corresponding protein sequences. This led to the generation of several thousand additional protein isoforms contained within a cell-specific alternate protein sequence database (Fig. 1*B*). Despite this large increase in the number of protein isoforms, the database search space increased by only 2%, which should result in no or very minor increase in false-positive identification during MS spectral search (37).

The alternate protein sequence database was then used, together with the reference database, for the processing of deep proteomics and phosphoproteomics data from A375 S and A375 R cells. High-resolution MS led to the identification of more than 7500 protein groups and 20,500 phosphorylated sites (Fig. 1*C*), of which over 5000 protein groups and 7000 phosphorylated sites were quantified in all samples (n = 6). Using the quantitative data, these replicates were also characterized by high positive correlations at both the proteome (range 0.95–0.98) and phosphoproteome (range 0.73–0.90) levels (supplemental Fig. S1*D*). Interestingly, a principal component analysis based on protein abundances revealed that the first dimension separated A375 R from A375 S replicates (Fig. 1*D*). On the second dimension, one of the A375 S replicates separated from the other replicates, which might be explained by a lower protein identification rate in that specific sample. The separation between A375 S and R was confirmed using the phosphorylated site abundances, although within the second dimension (supplemental Fig. S1*E*). These initial quality controls highlighted the high measurement reproducibility for this dataset, as well as the ability to discriminate two closely related cell lines.

### Key Signaling Pathways Are Perturbed in BRAFi-Sensitive and -Resistant A375 Cells

**Fig. 2:**
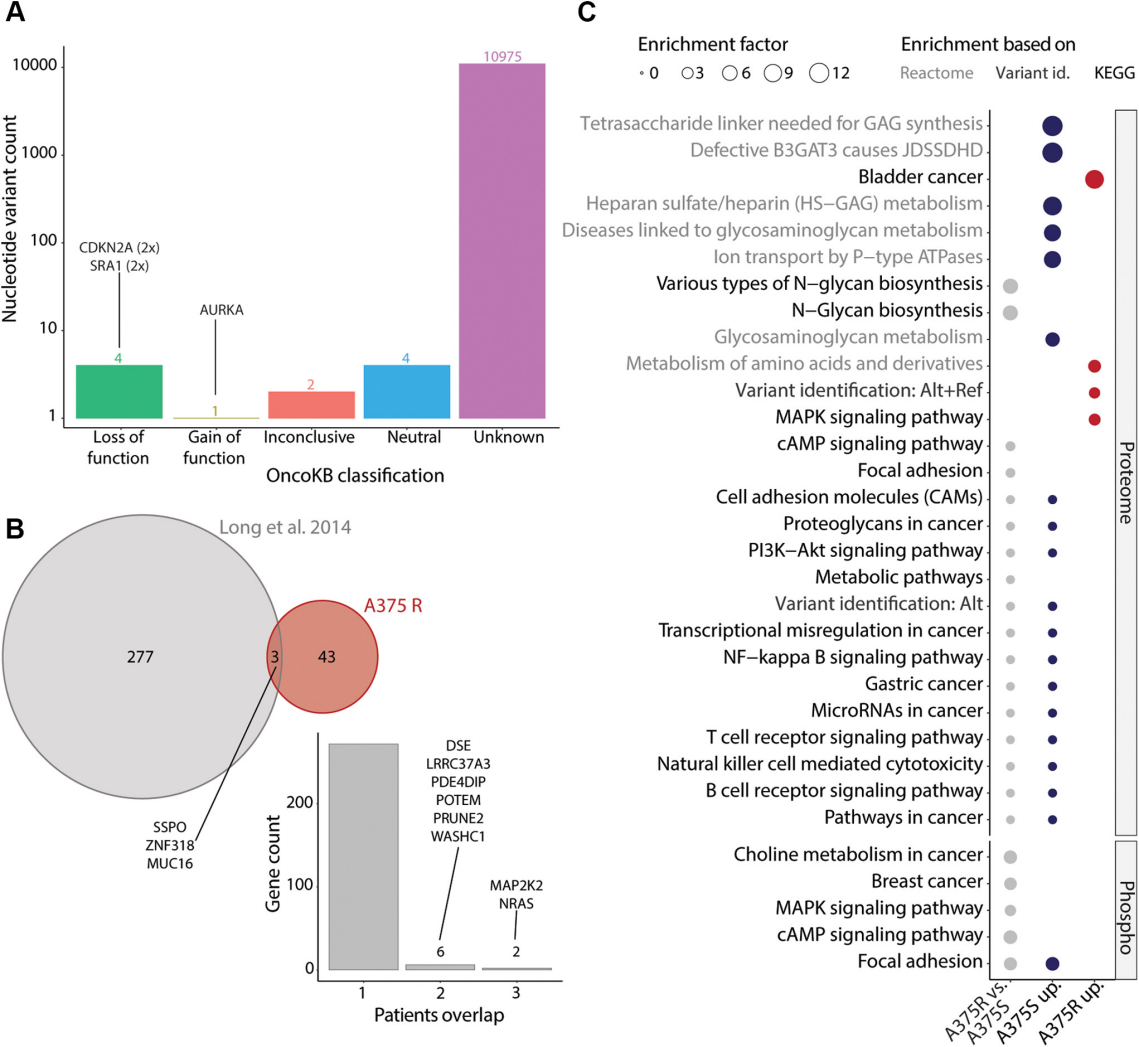
**Comparison of BRAFi-sensitive and -resistant A375 cells at the genome and (phospho)proteome levels.***A*, the nonsynonymous variants are counted per functional effect category as predicted using the oncoKB resource (38). *B*, a Venn diagram represents the overlap in genes with nonsynonymous variants between A375 R (unique variants identified in this study) and the patients from the study by Long *et al*. (29). Within the study from Long *et al.*, the genes with nonsynonymous nucleotide variants (found in resistant tumors) are represented as shared across one or more patients. *C*, the signaling pathways (KEGG and Reactome) are displayed based on their overrepresentation using differentially changing proteins or phosphorylated sites (Fisher exact test FDR ≤0.2). Pathway names are color-coded based on their functional database of origin (Reactome = *gray*, KEGG = *black*). The *size of the dot* represents the pathway enrichment factors. The *dot color* coding corresponds to overrepresentation using all significantly changing entries between A375 R and A375 S (*gray*), the entries that are significantly increased in A375 S (*blue*) or in A375 R (*red*).

Subsequently, we investigated the nonsynonymous nucleotide variants based on their functional effect as predicted by the oncoKB resource (38). This analysis revealed that five nonsynonymous nucleotide variants resulted in a possible loss of function for cyclin-dependent kinase inhibitor 2A (CDKN2A E61X) and steroid receptor RNA activator 1 (SRA1 V110RfsX24), as well as a possible gain of function for aurora kinase A (AURKA F31I) (Fig. 2*A*). These variants were reported as known somatic mutations in COSMIC in a range of cancer including melanoma. These were considered as potential driver mutations in context of resistance to BRAFi and retained for further analysis (39). To expand this driver list, we also overlapped the nonsynonymous nucleotide variants identified uniquely in A375 R against the study from Long *et al.* (29), who analyzed ten malignant melanoma patients (supplemental Table S2.1). While we did not observe any shared variants between A375 R and the patients' data, we did find three shared genes harboring different variants (Fig. 2*B*). Of note, the overlap in variants from resistant tumors found among the patients from the Long *et al.* study was also very limited, which led us to retain all A375 R unique variants as potential driver mutations for further analysis.

We next performed a quantitative comparison of A375 R *versus* A375 S using the protein and phosphorylated site abundances. MS analysis identified 359 significantly regulated proteins (*t* test with s0 at 0.1 and FDR ≤0.05) between cell lines (supplemental Fig. S2*A* and supplemental Table S1.2), including the BRAF resistance marker protein nestin (NES), which was identified in our previous study (23). At the phosphoproteome level, we identified 187 differentially abundant phosphorylated sites (*t* test with s0 at 0.1 and FDR ≤0.1) that were present on several key proteins, such as AP-1 transcription factor subunit (JUN) or eukaryotic translation initiation factor 4B (EIF4B) (supplemental Fig. S2*B* and supplemental Table S1.4). Deeper functional characterization was obtained through overrepresentation analyses based on different subset of significantly changing proteins and phosphorylated sites (Fisher exact test FDR ≤0.2). Notably the MAPK and cAMP signaling pathways, as well as focal adhesion, were overrepresented at both the proteome and phosphoproteome level (Fig. 2*C*, supplemental Tables S1.3 and S1.5). We also identified several other pathways connected to cancer, immune response, and glycoproteins (important for metastasis). Taken together, our data show that proteomics and phosphoproteomics deepen our understanding of the disrupted signaling pathways implicated in cell line model of BRAFi resistance.

### Amino Acid Variants Are Detectable within Key Signaling Pathways and Proteins

The aforementioned cell-specific protein database, used during the processing of the MS data, allowed us to detect a number of variant peptides, *i.e.*, peptides harboring reference or alternate amino acid. Among the MS-identified amino acid variants, most were shared across A375 R and S, a trend that was consistent at the proteome and phosphoproteome level (supplemental Fig. S2*C*). Interestingly, these MS-detected variant protein isoforms were overrepresented in cancer-related, immune response and glycoprotein pathways (supplemental Fig. S2*D* and supplemental Table S1.6). To confirm the quality of identification for these alternate variant peptides, we displayed the MaxQuant score, as well as intensity (mean and standard deviation) distribution, which was nearly identical to the rest of the peptides (supplemental Fig. S2, *E* and *F*). We also checked whether MS identification of amino acid variants was dependent on variant genetic zygosity. Our data shows that the majority of alternate variant peptides were homozygous based on WES; however, this trend did not translate to higher peptide intensities (supplemental Fig. S2, *G* and *H*).

Importantly, we could identify most of the commonly mutated genes in melanoma or in context of BRAFi resistance at the WES and/or (phospho)proteome levels (supplemental Fig. S2*I* and supplemental Table S1.7). However, only the BRAF V600 E variant could be identified at both the WES and proteome levels. Among the resistance marker genes found within the Long *et al*. study, only MAP2K3 (MEK3) harbored a variant in our WES dataset (R67W). These results highlight the capability of proteogenomics approach to detect amino acid variants on expressed proteins, which in turn reveal an overrepresentation of key cell signaling pathways.

### The Perturbed Signaling Network in BRAFi-resistant A375 Cells Can Be Targeted by Several Drugs

**Fig. 3:**
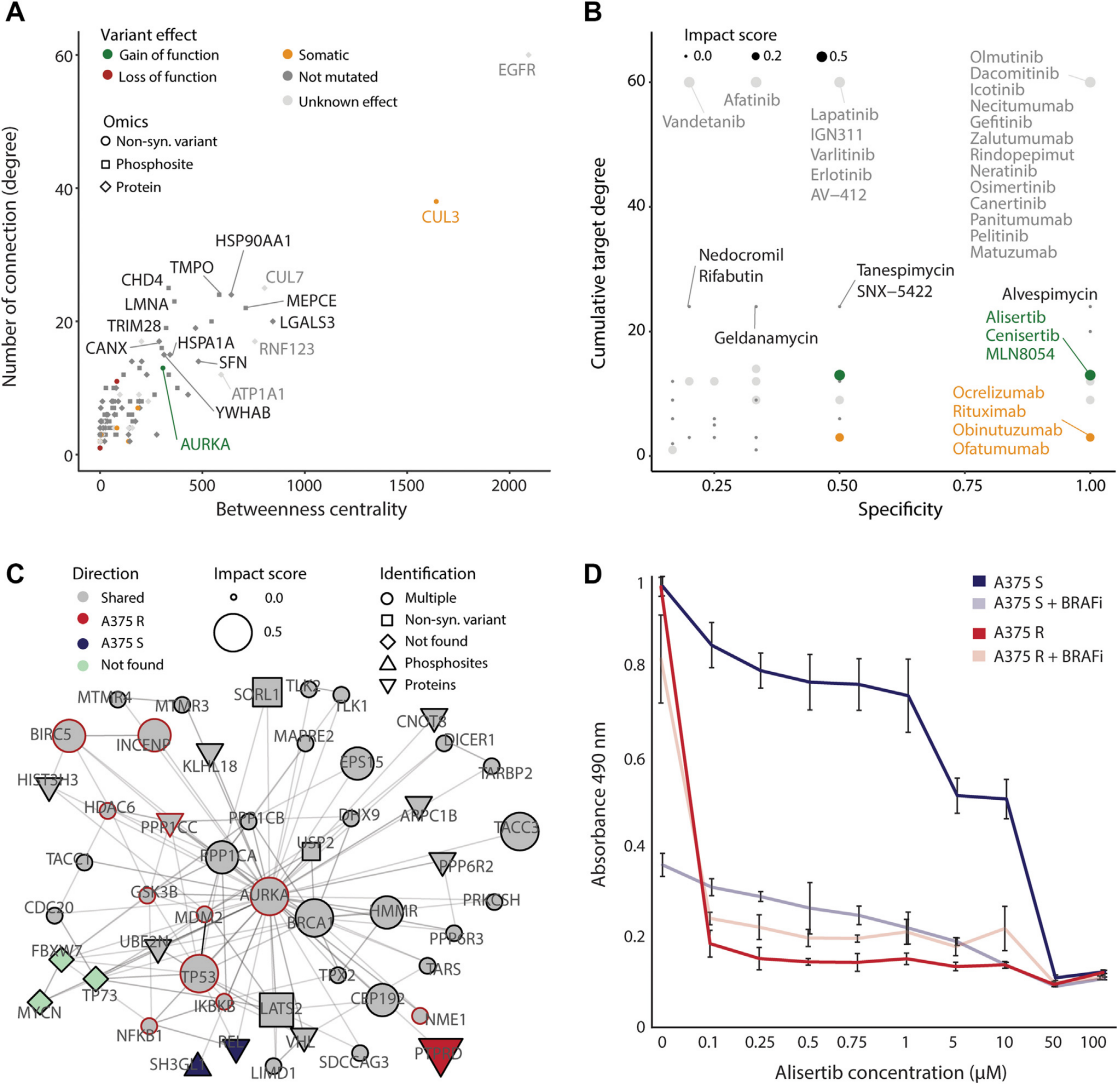
**The disturbed signaling network in BRAFi-resistant cells can be targeted by a number of drugs.***A*, the interaction signaling network is generated based on list of putative driver mutations (*circle*), proteins (*diamond*), and phosphorylation sites (*square*). This schematic displays the distribution of nodes in function of their betweenness centrality and number of connections. Only the top 200 entries are displayed (ranked based on their interaction degree). Entries are colored based on whether they harbor no variant (*dark gray*), a variant with unknown effect (*light gray*), a variant unique to A375 R (*orange*), a variant leading to loss of function (*red*) or a variant leading to gain of function (*green*). Entries that can be targeted by a drug are displayed with a *red* stroke. *B*, the drugs, interacting with entries from the interaction signaling network, are displayed based on their specificity to their target and how many connections their targets have. Color coding corresponds to whether one of the drug targets harbors no variant (*dark gray*), a variant with unknown effect (*light gray*), a variant unique to A375 R (*orange*), a variant leading to loss of function (*red*) or a variant leading to gain of function (*green*). *C*, the interaction signaling network allows visualization of the top 50 interactors (ranked based on their interaction degree) of AURKA. The shape of the nodes specifies whether the node was identified harboring a variant, quantified at the proteome level, quantified at the phosphoproteome level, found across more than one omics level, or not found. Entries are colored based on whether they were found significantly changing at the (phospho)proteome level, with increase abundance in A375 R (*red*) and A375 S (*blue*), not changing in abundance (*gray*), or not found (*light green*). Entries that can be targeted by a drug are displayed with a *red* stroke. The node size increases according to their importance in context of melanoma and BRAFi resistance (from 0 = no impact, up to 1 = high impact). *D*, cell viability assay of A375 S and A375 R cells treated with AURKA inhibitor alisertib at the indicated concentrations or in combination with the BRAF inhibitor vemurafenib (2 μM). Cell viability was determined with MTS assay 72h after treatment start (n = 6). Error bars represent standard deviations of replicates.

Subsequently, we integrated the significantly changing proteins and phosphorylation sites, as well as the list of potential driver mutations generated above, into a protein–protein interaction network (Fig. 3*A*, supplemental Fig. S3*A* and supplemental Table S1.8). Only the top 200 entries were retained on the basis of their number of connections obtained from the BioGRID database. The size of the entries is scaled up using a custom bioinformatic script to represent their importance in context of cancer, melanoma, and melanoma resistance (see Experimental Procedures). A list of drugs was retrieved from DrugBank database and their targets were marked within this network (supplemental Fig. S3*A*). Several entries were further highlighted through this approach due to their variant putative functional effect, such as AURKA (gain of function), CDKN2A and SRA1 (loss of function), CUL3, USP22, and MS4A1 (A375 R unique variants). Among these only AURKA and MS4A1 can be targeted by drugs, with AURKA displaying a relatively high betweenness centrality and number of degrees (Fig. 3*A*).

This allowed us to prioritize potential drugs based on their target specificity, as well as on the number of degrees their targets have with the rest of the signaling network (Fig. 3*B*). Among the prioritized therapies were inhibitors of EGFR (*e.g.*, olmutinib), HSP90AA1 (*e.g.*, alvespimycin), AURKA (*e.g.*, alisertib), and MS4A1 (*e.g.*, rituximab). Because AURKA was the only variant annotated with a gain of function, as well as a relatively large signaling network (Fig. 3*C*), we decided to experimentally validate the action of the compound alisertib on A375 R and S cells. We found that BRAFi-resistant A375 cells were sensitive to AURKA inhibition (AURKAi) with alisertib (40), regardless of the absence/presence of BRAFi vemurafenib (Fig. 3*D* and supplemental Fig. S3*B*). Conversely, A375 S tolerated alisertib (in the absence of BRAFi) and was able to proliferate. Our results demonstrate that the integration of proteogenomics datasets has the potential to predict effective and specific drug therapy in context of BRAFi resistance.

### Multiple Amino Acid Variants Directly Affect Protein Phosphorylation Status

**Fig. 4:**
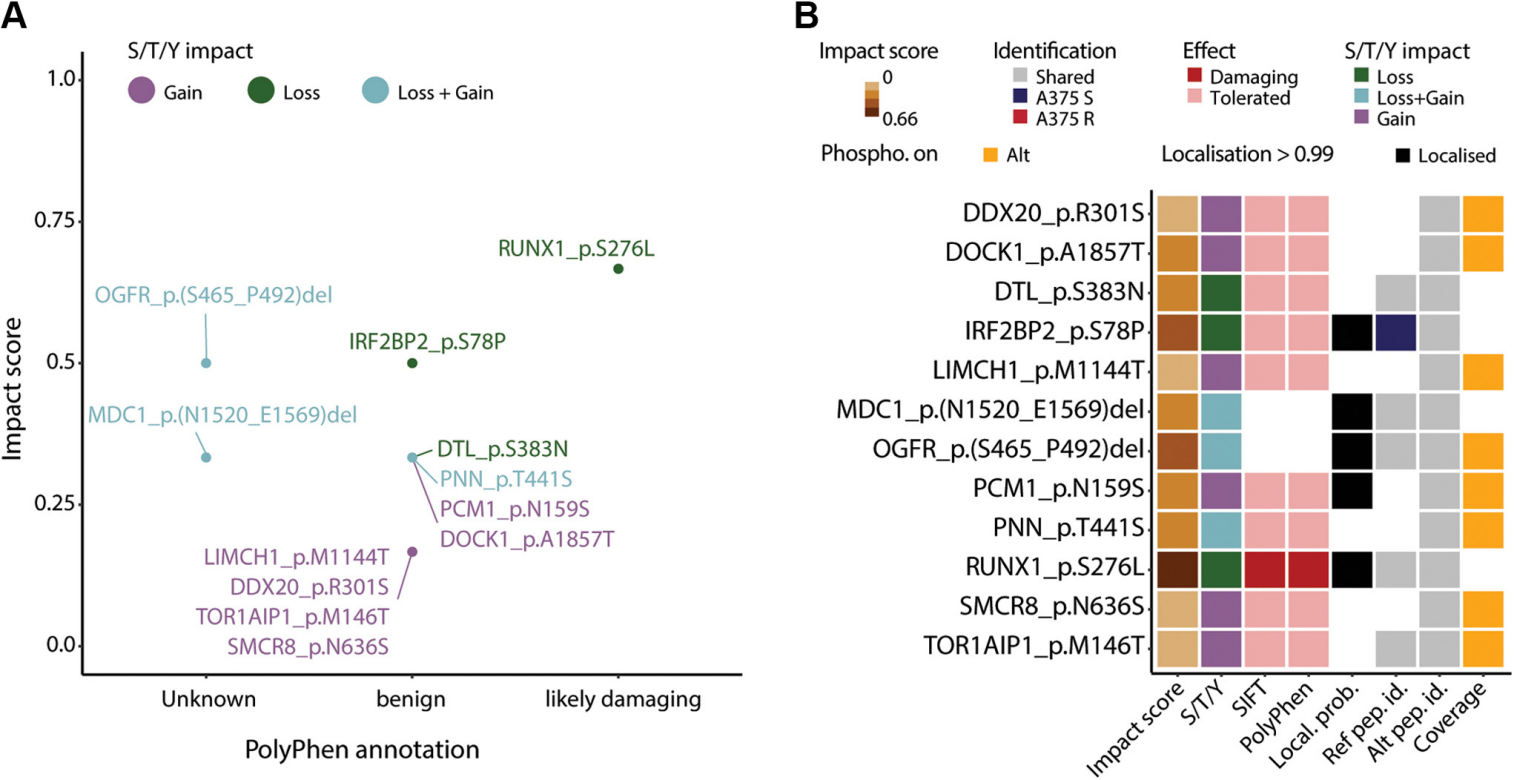
**Multiple amino acid variants directly affect protein phosphorylation status.***A*, scatter plot of the nonsynonymous amino acid variants that have an impact on protein phosphorylation status either as a loss of an S/T/Y amino acids (*green*), gain of S/T/Y (*purple*) or loss/gain of S/T/Y (*blue*). The PolyPhen annotation classifies these variants based on their possible effect on protein function, while the impact score prioritizes these variants due to their importance in context of melanoma and phosphorylation status disruption. *B*, heatmap of the nonsynonymous amino acid variants that have an impact of protein phosphorylation status. For each nonsynonymous amino acid variant is displayed its impact factor in context of melanoma and phosphorylation status, the actual phosphorylation effect (*i.e.*, loss, gain), the SIFT, and PolyPhen annotation, whether the modification was localized (*i.e.*, localization probability ≥0.99), whether the reference and alternate variant peptide were identified exclusively in A375 R or A375 S or in both, and whether the modification is found on the alternate variant peptide (as opposed to the reference).

Due to the elusive impact of amino acid variants on the phosphorylation status, we investigated all amino acid variants within A375 cells, irrespective of their role within BRAFi resistance. All nonsynonymous variants were annotated based on whether they had an impact on S/T/Y amino acids, kinase motifs, known variants, known PTMs, known oncogenes, and protein sequence changes superior to 90% of the reference protein (supplemental Fig. S4, *A* and *B* and supplemental Table S1.9). Interestingly, 6103 variants resulted in a loss of S/T/Y residues, whereas 5876 resulted in a gain, which represented a large potential for disrupting the phosphorylation-mediated cell signaling. Through investigation of the phosphoproteome dataset, we could confirm that 12 amino acid variants resulted in an actual loss or gain of phosphorylation events within A375 cells. To prioritize these variants, we annotated them using the PolyPhen software (as well as SIFT), which predicted a variant on RUNX1 protein (S276L) as likely damaging (Fig. 4*A*). Notably, this variant site on RUNX1 was one of the few variant sites detected with a high localization probability (Fig. 4*B*) and was reported in COSMIC as somatic in melanoma patient. To further evaluate the quality of MS identification, we compared the phosphorylated site intensities between the variant and all other sites, which revealed lower intensity among variant sites (supplemental Fig. S4*C*). Similarly, when displaying the MaxQuant score and localization probability, only a few phosphorylated variant sites (including the site on RUNX1) could be considered as high confidence sites (supplemental Fig. S4*D*). While these phosphorylated variant sites were not significantly changing in abundance between A375 R and S and thus are unlikely to be connected with BRAFi resistance, we hypothesized that they may still have a functional effect on A375 cell signaling.

### Loss of a Phosphorylation Site on RUNX1 Alters Its Interactome and Transcriptional Activity

**Fig. 5:**
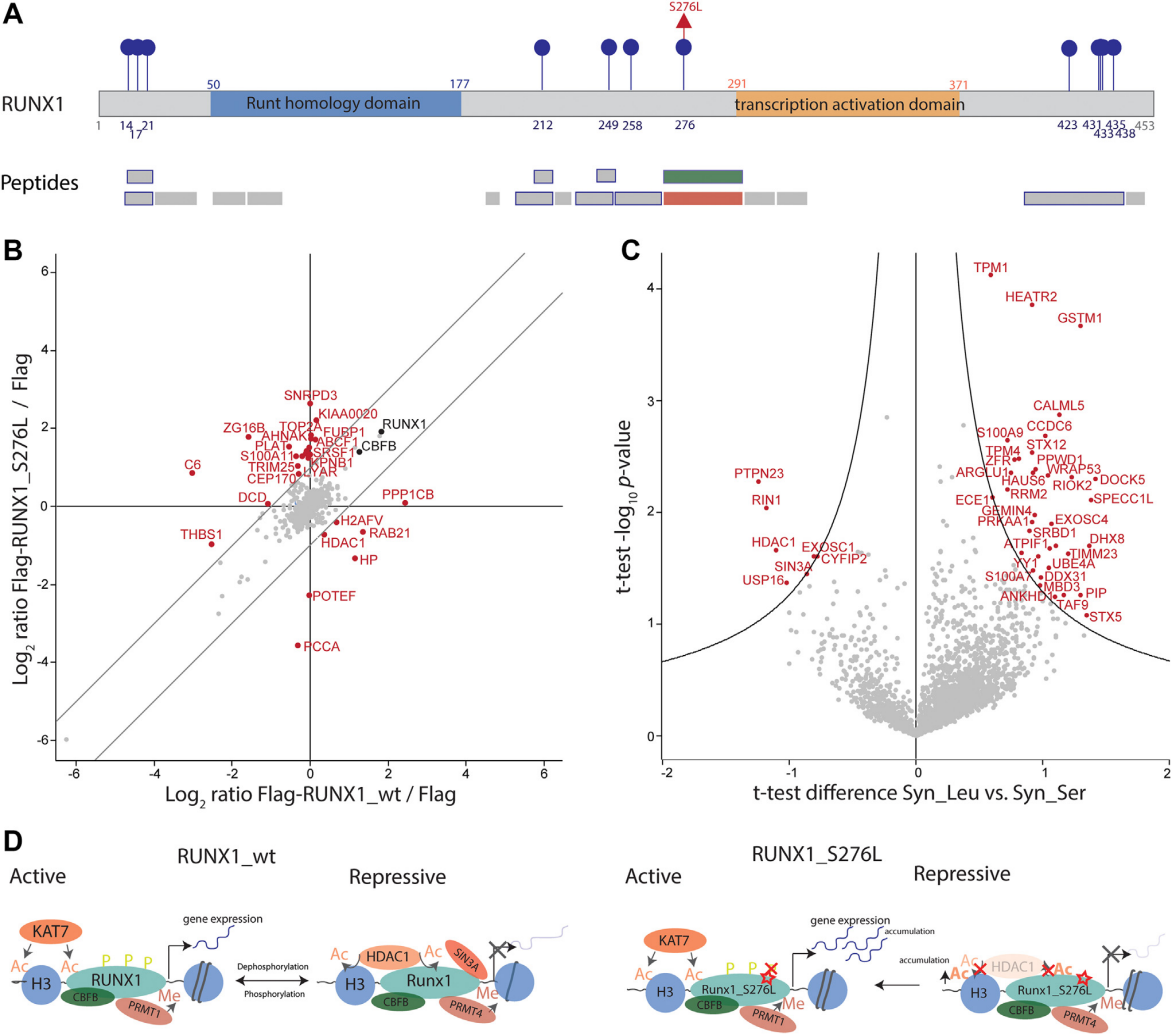
**Loss of a known phosphorylation site leads to a change in RUNX1 interactome.***A*, schematic overview of the transcription factors RUNX1 protein. *Numbers* indicate the positions of amino acids residues within the protein. Identified phosphorylation sites are highlighted in *blue* and identified amino acid variants are highlighted in *red*. Identified peptides by LC-MS/MS are shown in the second panel. Phosphorylated peptides are indicated with a *blue border*, while reference and alternate variant peptides are highlighted in *green* and *red*, respectively. *B*, interaction proteomics screen in A375 RUNX1_KO cells stably overexpressing Flag-tagged RUNX1_wt or Flag-tagged RUNX1_S276L. SILAC protein expression (log_2_) of RUNX1_wt or Flag-tagged RUNX1_S276L relative to the corresponding control cell line (Flag tag only). RUNX1 and its core binding factor CBFB are marked in *black*. Significantly up and downregulated proteins are highlighted in *red*. Results represent three replicates per experiment group. *C*, volcano plot of synthetic alternate peptide (Syn_Leu) *versus* synthetic reference peptide (Syn_Ser) pull-downs of A375 cells. The log_2_ fold change in abundance between Syn_Leu and Syn_Ser is plotted against −log_10_*p*-value (n = 3). *Black lines* indicate the significance threshold based on Student's *t* test (FDR <0.01; s0 = 1.2). Significantly up and downregulated proteins are highlighted in *red*. *D*, schematic overview of proposed interaction of RUNX1_wt and RUNX1_S276L with main transcriptional regulators.

We then focused on the variant impacting the phosphorylation status of RUNX1 protein, a key transcription factor involved in cell proliferation, differentiation, and apoptosis (36). This amino acid variant led to the loss of a known phosphorylation site due to change from serine to leucine at position 276 (Fig. 5*A*). The reference and alternate variant peptides were identified with high resolution MS in both A375 S and R cells (supplemental Fig. S5, *A* and *B*). We hypothesized that this variant is likely to influence the interactome of RUNX1. Therefore, we generated an RUNX1 gene knockout in A375 S cells using the CRISPR/Cas9 system. Single cell clones were selected for further analysis based on their effective RUNX1 knockout (KO). As a control we used a nontargeting (NonTar) control guide sequence. A375 RUNX1 KO cells showed an insertion of 215 bp in the Exon 1 of the gene compared with reference DNA of A375 S cells (supplemental Fig. S5*C*). The lack of expression of RUNX1 protein was confirmed by western blot and MS analysis (supplemental Fig. S5*D*). To study the impact of the loss of a modifiable amino acid, we performed immunoprecipitation of Flag-tagged RUNX1_wt and RUNX1_S276L in RUNX1 KO SILAC-labeled cells in three independent replicates (Fig. 5*B*, supplemental Tables S3.1 and S3.2). The interactome analysis by LC-MS/MS revealed that RUNX1 and its core binding factor CBFB were significantly enriched in both pull-downs compared with Flag-empty vector (supplemental Fig. S5, *E* and *F*). Interestingly, the known interaction partner histone deacetyltransferase HDAC1 was enriched in RUNX1_wt interactome and depleted in the RUNX1_S276L interactome (Fig. 5*B*).

To assess the impact of this variant on the interactome, we performed pull-down assays with synthetic peptides harboring the amino acid sequence for reference and alternate variant of RUNX1 in A375 cells (Fig. 5*C*, supplemental Tables S3.3 and S3.4). As in the interactome study, HDAC1 was significantly depleted in the pull-down of alternate *versus* reference variant peptide indicating that the interaction between HDAC1 and RUNX1 is disturbed due to the variant. We also identified transcriptional repressor SIN3A to be significantly depleted in alternate variant peptide pull-down compared with reference pull-down similar to HDAC1. RIN1 and PTPN23 showed the same trend as HDAC1 and SIN3A; and both proteins are known to act as regulator of RAS-mediated mitogenic activity (41, 42). The proteins enriched in alternate pull-down compared with reference variant peptide pull-down were overrepresented in TGFβ signaling, melanogenesis, and insulin signaling pathways (supplemental Fig. S5*G* and supplemental Table S3.5). Taken together, we demonstrate that the loss of the known phosphorylation site on S276 has an impact on the interactome of RUNX1 (supplemental Fig. S5*H*) and postulate that it leads to altered transcriptional activity of the protein (Fig. 5*D*).

## Discussion

In this study, we used a common cell line model of melanoma (A375), a well-known cancer for its high mutation load (1) and the potential for rewiring cellular networks (16, 43). While cell lines have shown to adequately retain the driver mutations at the genomic level, they do not recapitulate the extent of patient or tumor heterogeneity. In addition, high-throughput transcriptomic and proteomic studies have shown that cell lines differ from their tissue of origin and may be of limited use as model (44). Two consortia, namely the Clinical Proteomic Tumor Analysis Consortium and The Cancer Genome Atlas, have greatly contributed to the development of onco-proteogenomics applied to cell line and patient material (11, 12, 13). However, proteogenomics studies are still relatively rare and, due to their complexity, out of reach of most proteomics (or genomics) laboratories. Here, we use such a proof-of-concept proteogenomics workflow to analyze a single melanoma cell line in order to (1) predict sample-specific drug therapies in context of BRAFi resistance and to (2) investigate variant impact on phosphorylation-mediated cell signaling irrespective of BRAFi resistance.

### Proteogenomics Reconstructs the Signaling Network Linked to BRAFi Resistance in A375 Cells

In this study, the nucleotide variant analysis revealed very similar numbers across A375 cell lines, the large majority being SNVs, which is consistent with a previous study (45). We also observed characteristic nucleotide substitutions, whereby two-thirds of substitutions are comprised of transitions. The C to T transition was highly represented and is known to result from sunlight exposure, which is highly relevant for skin cancer (46). The proteome coverage we obtained for A375 cells is similar to other state-of-the-art MS-based study of cancer cell lines (47). The differentially changing proteins and phosphorylated sites between A375 R and S revealed that the MAPK signaling, cAMP signaling, and focal adhesion pathways were found overrepresented and are of critical importance for melanoma resistance to BRAFi (48).

We then reconstructed the signaling network associated with BRAFi resistance in A375 cells; *i.e.*, using the putative driver mutations, as well as the differentially abundant proteins and phosphorylation sites. This approach highlighted several hubs and high impact entries, such as (1) variants leading to gain of function on AURKA, loss of function on CDKN2A and SRA1 and A375 R unique variants on CUL3, USP22, and MS4A1; (2) increase in protein abundance within A375 R for EGFR, and increased within A375 S for HSP90AA1; and (3) increase in phosphorylated sites abundance within A375 S for JUN. Among these entries, several are known for their involvement in melanoma susceptibility, development, resistance, and therapy (49, 50, 51, 52, 53, 54, 55). Several drugs were identified and ranked based on their potential to disrupt this signaling network; notably alisertib, a highly specific inhibitor of AURKA, which has been previously reported for its beneficial effect in combination with BRAF and MEK inhibitors in melanoma treatment (40, 49). We experimentally validated the use of alisertib on A375 S and R cells and could show that A375 R cells are sensitive to AURKAi. Our data confirm that AURKA has a critical role in the context of resistance and may be suitable for the treatment of melanoma as reported previously (56

### Proteogenomics Pinpoints Several Peptides That Are Phosphorylated on the Variant Site

We investigated further the amino acid variants that could be confirmed by MS and may be important in melanoma development or resistance. Over 500 protein groups and 158 phosphorylated sites were found with at least one alternate variant peptide. Our identification results are in the same range (or higher) as other studies investigating amino acid variants using custom protein sequence databases (57, 58). Interestingly, an overrepresentation analysis of alternate variant proteins revealed cancer-related, immune response and glycoprotein pathways (59, 60, 61, 62, 63). There are two possible explanations for such a result; either it suggests an accumulation of variants on proteins belonging to these pathways, as these variants would provide a survival advantage for cancerous cells. Or the proteins harboring these variants have inherent characteristics that facilitate their detection by MS (*e.g.*, protein length), thus facilitating amino acid variant detection even if these variants do not contribute to cancer development or BRAFi resistance (39, 58).

As observed in our dataset, many variants may have an impact on the PTM of proteins. Around 14.8% of all amino acids in the human proteome are serine, threonine, or tyrosine (28), which are predominantly modified by phosphorylation. Several studies have reported that these three amino acids are disproportionally affected by missense mutations (64, 65). While these may not all be relevant in tumor cells, since not all genes are expressed at any one time, previous studies have shown the deleterious effect of such variants (12, 16). Here, we confirmed by MS a change in phosphorylation status for 12 variant protein isoforms, either as a loss or gain of phosphorylated sites. Several of these protein isoforms were key cell signaling molecules and may be involved in cancer development, for example, MDC1 (66), OGFR (67) and PCM1 (68). Interestingly, we also confirmed by MS the presence of a variant on RUNX1, which was annotated as likely damaging due to its known role in cancer and loss of phosphorylation site at position S276 (69). However, in this study, the variants affecting protein modification status were shared across A375 S and R and did not show significant change in abundance, suggesting that they are not involved in BRAFi resistance mechanisms of A375 cells. The identification of these variants by MS is worth noting, and further work is needed to increase the identification score and localization probability of the alternate variant peptides, as well as to investigate their function.

### Rewiring of Signal Transduction Network Due to Loss of a Known Phosphorylation Site on RUNX1

We experimentally validated this striking example of a loss of a known phosphorylation site on RUNX1 and showed that this variant has an impact on the interactome of RUNX1. The transcription factor RUNX1 is mutated in 3.03% of melanoma patients, and so far, 43 mutations are described in the literature for cutaneous melanoma (70). The variant site S276L of RUNX1 is located in a highly modified region of the protein and may influence the nearby transcriptional activation domain. Wee *et al.* (69) showed *in vitro* that the triple phosphorylations at the sites S249, T273, and S276 are important for the interaction with the histone acetyltransferase p300 and thus lead to the regulation of gene transcription *via* chromatin remodeling. Here, we could not identify p300 in the interactome studies of RUNX1 by immunoprecipitation of overexpressed RUNX1 or synthetic peptide pull-downs. However, we identified the transcriptional activator WWTR1 (TAZ) and KAT7 and the corresponding transcriptional repressors HDAC1 and SIN3A to be changing between reference and alternate pull-down of RUNX1. The loss of the interaction to HDAC1 by mutating RUNX1 at S48, S303, and S424 to aspartic acid *in vitro* has been described previously (71). Here, we hypothesized that the interaction is associated with the modification status of the protein. The cross talk between acetylation/deacetylation-mediated and phosphorylation/dephosphorylation may alter the transcriptional activity by RUNX1. It is well known in the literature that RUNX1_wt switches between active and repressive state due to modifications such as acetylation and phosphorylation and binding of interaction partners such as HDAC1, PRMT1, and P300 (72). We postulate that RUNX1_S276L remains in the active state due the loss of binding to transcriptional repressors such as HDAC1 and SIN3A, which could lead to the accumulation of acetylation on the protein itself as well as histones. This may result in stronger transcriptional activity, which should be tested in further experiments. Taken together, we propose that this variant changed the interactome of RUNX1 and altered the transcriptional activity of RUNX1.

## Conclusion

Proteogenomics is a powerful tool to study the mode of action of disease-associated mutations at the genome, transcriptome, proteome, and PTM level. Here, we applied a proteogenomics workflow to study the melanoma cell line A375 sensitive and resistant to BRAF inhibition. The investigation and integration of multiomic datasets allowed us to reconstruct the perturbed signaling networks associated with BRAFi resistance of A375 cells. This resulted in the prioritization of key actionable nodes and the prediction of drug therapies with the potential to disrupt BRAFi resistance mechanism in A375 cells. Notably, we demonstrated the use of AURKA inhibitor as an effective and specific drug against BRAFi-resistant A375 cells. We also detected the loss or gain of several phosphorylation events due to variants. We could confirm the loss of Ser276 phosphorylation site by MS as a direct consequence of variant S276L on the transcription factor RUNX1. Our results suggest that this mutation has an impact on the interactome of RUNX1 and may be responsible for change in its transcriptional activity. Such proteogenomics workflow, used here as a proof of concept on A375 cells, may be applicable to other types of cancer, cell lines, or even patient-derived samples.

## Data availability

The high-throughput nucleotide sequencing data have been deposited in the NCBI Sequence Read Archive (73) with the bioproject accession number PRJNA616103. The mass spectrometry proteomics data have been deposited to the ProteomeXchange Consortium *via* the PRIDE (74) partner repository with the dataset identifier PXD018305. Visualization of MS/MS spectra is possible at https://msviewer.ucsf.edu/prospector/cgi-bin/msform.cgi?form=msviewer *via* the search keys: lixu3zlmbb (comparison of A375 S *versus* A375 R), livyy138s (RUNX1 overexpression), and tztqlaxjtr (RUNX1 peptide pull-down). The WES bioinformatics pipeline is available online (75).

## Supplemental data

This article contains supplemental data (29, 38, 76, 77, 78, 79, 80, 81).

## Conflict of interest

The authors declare no competing interests.
